# Gastric Cancer Associated Genes Identified by an Integrative Analysis of Gene Expression Data

**DOI:** 10.1155/2017/7259097

**Published:** 2017-01-23

**Authors:** Bing Jiang, Shuwen Li, Zhi Jiang, Ping Shao

**Affiliations:** ^1^Department of Spleen and Stomach Diseases, Hospital of Traditional Chinese Medicine, Yixing, Jiangsu 214200, China; ^2^Department of Gastroenterology, The First Affiliated Hospital of Soochow University, Suzhou, Jiangsu 215006, China; ^3^Department of Biochemistry and Molecular Biology, School of Medicine, Soochow University, Suzhou, Jiangsu 215123, China

## Abstract

Gastric cancer is one of the most severe complex diseases with high morbidity and mortality in the world. The molecular mechanisms and risk factors for this disease are still not clear since the cancer heterogeneity caused by different genetic and environmental factors. With more and more expression data accumulated nowadays, we can perform integrative analysis for these data to understand the complexity of gastric cancer and to identify consensus players for the heterogeneous cancer. In the present work, we screened the published gene expression data and analyzed them with integrative tool, combined with pathway and gene ontology enrichment investigation. We identified several consensus differentially expressed genes and these genes were further confirmed with literature mining; at last, two genes, that is, immunoglobulin J chain and C-X-C motif chemokine ligand 17, were screened as novel gastric cancer associated genes. Experimental validation is proposed to further confirm this finding.

## 1. Introduction

Gastric cancer (GC) is one of the most severe cancers in the world with high incidence and low survival rate. According to the global cancer statistics report in 2012, GC has been the fifth most common cancer in the world, which causes more than seven hundred thousand deaths each year [1]. Usually, the number of GC patients in men is twice more than that in women and Eastern Asia, especially Korea, Japan, and China, has the highest incidence rate. Although relevant reports revealed that the age-standardized incidence rate of gastric cancer is decreasing in Japan and Korea in last few years [2, 3], the number of new cases is still increasing due to the aging of the population. The pathogenesis of gastric cancer is very complex and remains unclear. Recent basic studies mainly focus on three main factors: environmental factors,* Helicobacter pylori* (*H. pylori*) infection, and gene expression dysregulation [4, 5]. Previous studies have demonstrated the unhealthy lifestyle, such as excessive diet, can raise the risk of gastric cancer [5–7]. Processed meat intakes will increase the risk of gastric non-cardia cancer in* H. pylori* antibody-positive individuals while fresh fruits and vegetables consumption will protect individuals against GC. Also, in molecular level, several host genetic factors might play a key role in GC, such as IL-1*β*, IL-10, TFF2, and CDH1 [8–10].

With relevant studies deepening, the size of research data is becoming larger and larger. Hundreds of gene expression profiles and diagnostic targets are uploaded into various gene expression databases. These data can be further integrated to the understanding of the complexity of the diseases, such as the cancer heterogeneity, high level consensus [11–13], biomarker discovery [14, 15], and the key players in the cancer genesis and progress [16]. In this study, we used meta-analysis approach for analysis of multiple transcriptomic datasets. We hope to integrate different gene expression data collected from GC patients and normal controls to figure out robust candidates in genes, pathways, and functions, setting the foundation for personalized treatment of gastric cancer.

The method we used here was named INMEX (integrative meta-analysis of expression data) program [17]. Data procession and screening were performed in order to make sure all the datasets we uploaded into the program were in a consistent format. Due to the existence of outliers and variations in microarray data, a combining rank orders algorithm based on RankProd package [18] was used here to carry out the meta-analysis.

## 2. Materials and Methods

**Figure 1:**
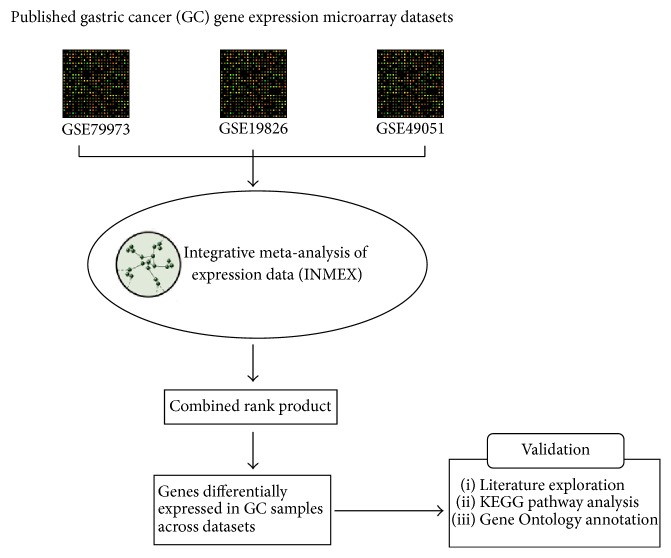
The pipeline of the whole analysis in this study.

The pipeline of this whole analysis in the present study is shown in Figure 1. We first extract the microarray gene expression data from the GEO database, then we integrate analyzed the expression data with a meta-analysis tool INMEX, and then we further screen and validate the meta-analysis results with literature analysis and bioinformatics functional analysis.

### 2.1. Dataset Collection and Data Screening

We used keywords “gastric cancer,” with two filters: (a) organism:* Homo sapiens* and (b) type: expression profiling by array, in searching for the gene expression profiles in Gene Expression Omnibus (GEO) database. We explored the searching results by setting four inclusion criteria: (1) datasets published after 2010; (2) case-control studies; (3) sample numbers more than 20; (4) high similarity in sample background information (i.e., sources, patients' race and location, disease status, and platforms). Datasets meeting these criteria were selected for further analysis.

### 2.2. Meta-Analysis for Selected Datasets

Based on the expression data we collected from each qualified microarray study, a global meta-analysis for identifying differentially expressed (DE) genes in gastric genes was conducted in this study. Here, we selected a web-based tool named INMEX (integrative meta-analysis of expression data, http://www.networkanalyst.ca/) for meta-analysis.

We firstly upload the normalized gene expression datasets into INMEX. Then we processed and annotated the datasets to adjust the data format and class labels into the consistent style. After the integrity check, we selected combining rank orders method, which is based on the RankProd package, to carry out the meta-analysis. The number of permutation tests in this method was 20 times.

### 2.3. Functional Enrichment Analysis of DE Genes

Functional enrichment analysis of these DE genes was further performed by INMEX program in two approaches: Geno Ontology and pathway analysis. In GO annotation, we set a *p* value threshold of 0.05 to identify the significantly enriched items. In pathway analysis, KEGG pathway database was used here for pathway enrichment analysis. A *p* value threshold of 0.05 was also set for identification of significantly enriched pathways.

## 3. Results

### 3.1. Characteristics of Datasets Included in This Meta-Analysis

**Figure 2:**
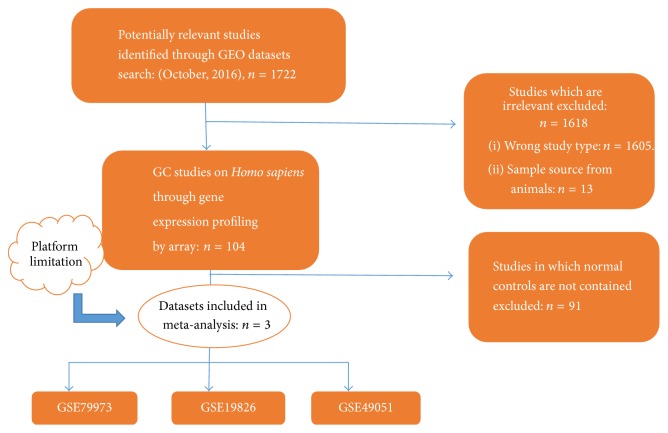
Datasets selection strategy and results.

The datasets selection strategy and the screening results are presented in Figure 2. Through GEO datasets searching, a total of 1722 studies were retrieved. 1618 irrelevant studies were excluded, among which 1605 studies were not expression profiling by microarray technologies and 13 studies were animal studies. The remaining 104 studies were included for full-text review. Studies without case-control matches were then excluded. Due to the platform limitation, we further excluded those studies whose microarray platforms are not available in INMEX program. After several rounds of screening, a final list of 3 microarray datasets [19, 20] was selected for meta-analysis.

These 3 datasets (GSE79973, GSE19826, and GSE49051) contain totally 25 cases and 25 controls. The number of cases and controls of each dataset is well matched. All the datasets were collected from Chinese hospitals and sample sources are consistent. The detailed information of these 3 datasets is listed in Table 1.

### 3.2. Results of Meta-Analysis

This study is performed based on combining rank orders. DE genes with *p* value < 0.05 were selected. Totally 1153 DE genes were got through this meta-analysis. The detailed DE gene information was listed in Table S1 (see Supplementary Material available online at https://doi.org/10.1155/2017/7259097). All of these DE genes are those identified to be differentially expressed in these three datasets rather than in individual samples. Among the 1153 DE genes, 787 genes were downregulated and 366 genes were upregulated.

The top 10 most significantly upregulated genes and top 10 most downregulated genes were listed in Tables 2 and 3. Genes with the smallest combined rank product (RP) in upregulated DE gene list and downregulated DE gene list are COL6A3 (combinedRP = 59.02) and PGC (combinedRP = 22.38), respectively.

### 3.3. Functional Enrichment Analysis Results

**Figure 3:**
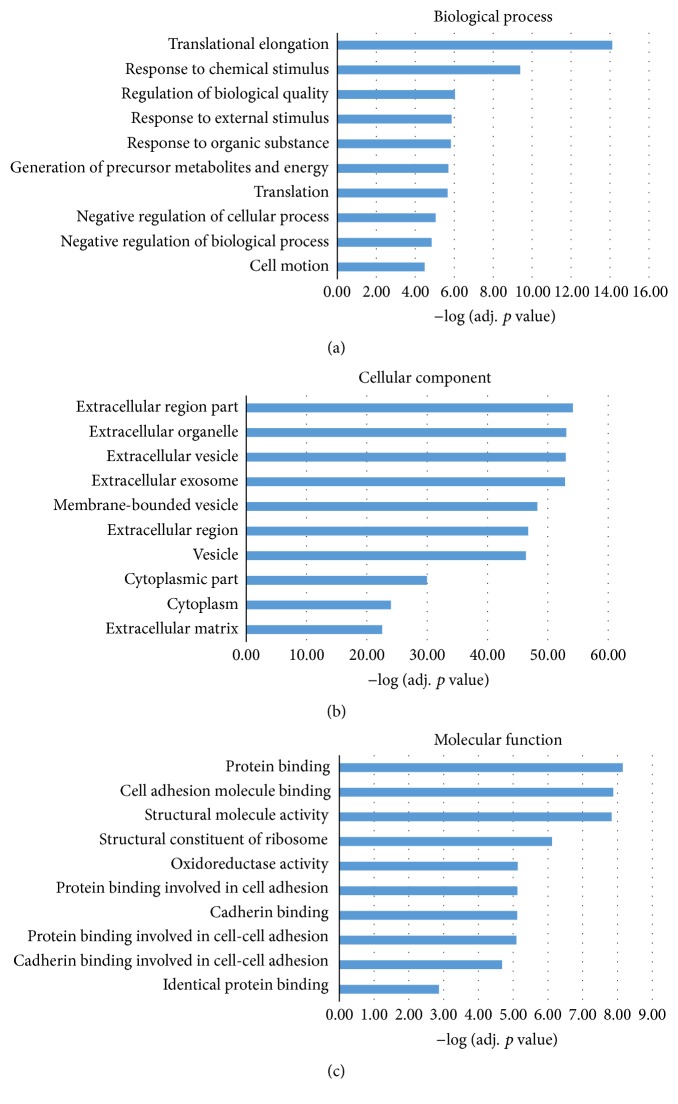
Gene Oncology (GO) annotation for the DE genes in gastric cancer. Here the GO annotation was used at three levels: biological process, cellular component, and molecular function. (a), (b), and (c) represent the top 10 most significantly enriched GO terms for these DE genes, respectively. All the adjusted statistical significance value (*p* value) of the terms was negative 10-based transformed.

**Figure 4:**
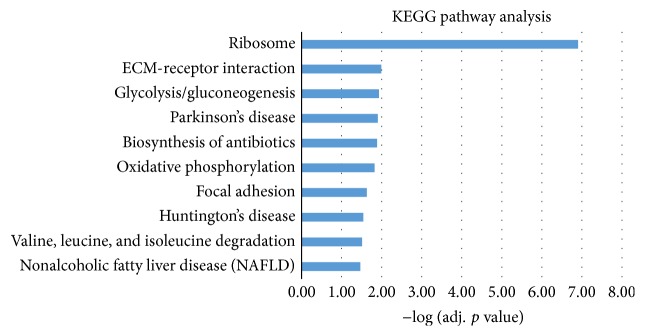
The top 10 most significantly enriched pathways in KEGG pathway analysis for the DE genes in gastric cancer. The adjusted statistical significance value (*p* value) was negative 10-based log transformed.

Functional enrichment analysis was carried out for further study of these DE genes. Gene Oncology (GO) analysis and KEGG pathway analysis were the two approaches we conducted here. In GO analysis, we did the analysis at three levels: biological process (BP), cellular component (CC), and molecular function (MF). The top 10 most significantly enriched terms (adj. *p* value < 0.05) were selected, respectively. The histograms of these terms were shown in Figure 3. Most of the DE genes are well mapped onto gastric cancer associated process of biological factors. In KEGG pathway analysis, we also selected top 10 most significantly enriched pathways, as shown in Figure 4. All of the selected items were taken into literature validation for further investigation.

## 4. Discussion

In this study, we have used publicly available microarray datasets to identify genes that are differentially expressed in tumor tissues from people with GC comparing to people without GC. The aim of our study is to derive additional information from the combining datasets that are unlikely to be established from individual studies in isolation through combining the data from three separate gene expression datasets in a meta-analysis. Generally, we found this is to be the case. Through PubMed literature mining, we found 8 of 10 of downregulated genes and all the upregulated genes have been reported to be associated with gastric cancer by biological and clinical experiment validation. For example, downregulated gene with smallest combinedRP in this study is Progastricsin (PGC). Many researchers have found it plays a key role in gastric cancer and the PGC polymorphism could serve as one of the diagnosis biomarkers for GC [21–23]. Also, in a recent research, Li et al. found, in mitogen-activated protein kinase activator with WD40 repeats (MAWD) and MAWD-binding protein (MAWBP) downregulated GC cells, the expression level of PGC was lower than that in control samples [24]. In upregulated genes, collagen VI *α*3 (COL6A3) is the gene with smallest combinedRP. Relevant research has found the expression level of COL6A3 was significantly higher in GC patients [25, 26], which also could serve as a diagnosis biomarker for GC. Other DE genes, such as COL1A2 [26], OLFM4 [27], THBS2 [28], CEACAM6 [29], CTSE [30], AKR1B10 [31], and KCNE2 [32], also have been reported to be differentially expressed in GC patients comparing to controls.

Interestingly, in the top 10 downregulated DE genes, 2 genes (IGJ and CXCL17) have not been reported to have a direct association with GC. For IGJ, Tvarijonaviciute et al. have observed that, in obese dogs, the amount of IGJ proteins was decreased [33]. Relevant research has revealed that obesity will increase the risk of gastric cancer [34]. For CXCL17, it is reported that overexpression of CXCL17 has a strong connection with colon cancer and hepatocellular carcinoma [35, 36]. The existence of gene interaction reveals the association between GC and these two cancers [37, 38]. Because there are still no specific experiments on these two genes and GC, further biological and clinical research are needed.

To further investigate the functional mechanisms of these DE genes, we performed GO analysis and KEGG pathway analysis. We finally get 102 significantly enriched terms (*p* value < 0.05) in biological process level, 157 in cellular component level, and 31 in molecular function level. As shown above, the top 10 significantly enriched terms were all reported to be associated with GC. For example, in extracellular matrix, there exists extracellular matrix protein 1 (ECM1). ECM1 plays a key role in lymphangiogenesis [39], which could be an inducement of cancer invasion and metastasis. Aberrant expression of ECM1 was found in GC samples in a recent study [40]. Also, in translational elongation process, relevant genes, such as translation elongation factor EEF1B2, were upregulated in the poor prognosis samples [41]. All the top 10 terms in BP, CC, and MF have been reported to have an association with GC.

In KEGG pathway analysis, the most significantly enriched pathway is Ribosome. Genes such as RPL11, RPL23, RPS6, and MRPS21 were enriched on this pathway. Ribosomal protein family (PRL/RPS) has been demonstrated to have a strong connection with GC. For example, a recent study revealed that GLTSCR2 regulates the MDM2-TP53 pathway through RPL11, playing a key role in GC progression [42]. A previous study has observed that reducing the phosphorylation of RPS6 could have an influence on the sensitivity to MEK inhibition in gastric cancer cells [43]. Another important pathway in GC is glycolysis/gluconeogenesis pathway. Reports revealed that microRNA-133b could silence PKM-splicer PTBP1, leading the inhibition of growth of human gastric cancer cells [44]. Hu and Chen also found that SIRT3 can strengthen glycolysis in SIRT3-expressing GC cells. Other pathways, like ECM-receptor interaction and metabolism of xenobiotics by cytochrome P450, have been validated to be associated with GC through bioinformatics approaches based protein-protein interaction networks analysis [45].

## 5. Conclusions

To summarize, our research provides novel angels in pathogenesis of gastric cancer. We identified consistently DE genes in gastric cancer through INMEX meta-analysis tools. Top 10 of upregulated and downregulated genes could potentially serve as diagnosis biomarker. GO annotation and KEGG pathway analysis demonstrated those candidates have a strong relationship with gastric cancer. Moreover, we identified 2 novel GC associated genes, IGJ and CXCL17, which have never been reported to be associated with GC before. Further experimental validation should be conducted in order to understand the mechanism of these two genes on gastric cancer.

## Supplementary Material

Table S1: Information for differentially expressed genes.

## Figures and Tables

**Table 1:** Datasets selected in this meta-analysis.

Accession/ID	Platform	GC	Control	Materials	Year	Race	Region
GSE79973	GPL570	*n* = 10	*n* = 10	Gastric tissues	2016	Chinese	Hangzhou
GSE19826	GPL570	*n* = 12	*n* = 12	Gastric tissue	2010	Chinese	Shanghai
GSE49051	GPL10332	*n* = 3	*n* = 3	Gastric tissue	2013	Chinese	Shanghai

**Table 2:** Top 10 most significantly downregulated DE genes in gastric cancer.

EntrezID	Gene full name	Gene symbol	CombinedRP	AveLogFC
5225	Progastricsin	PGC	22.38	−14454.48
57016	Aldo-keto reductase family 1 member B10	AKR1B10	22.86	−6705.56
9992	Potassium voltage-gated channel subfamily E regulatory subunit 2	KCNE2	36.08	−4314.33
284340	C-X-C motif chemokine ligand 17	CXCL17	49.18	−3880.57
135656	Diffuse panbronchiolitis critical region 1	DPCR1	55.94	−1892.04
51208	Claudin 18	CLDN18	57.35	−3435.26
3512	Immunoglobulin J polypeptide, linker protein for immunoglobulin alpha, and mu polypeptides	IGJ	62.81	−25978.05
1510	Cathepsin E	CTSE	64.51	−4631.28
340547	V-set and immunoglobulin domain containing 1	VSIG1	69.66	−1752.54
4499	Metallothionein 1M	MT1M	100.58	−6787.14

**Table 3:** Top 10 most significantly upregulated DE genes in gastric cancer.

EntrezID	Gene full name	Gene symbol	CombinedRP	AveLogFC
1293	Collagen type VI alpha 3 chain	COL6A3	59.02	3600.96
1278	Collagen type I alpha 2 chain	COL1A2	62.06	3576.21
10562	Olfactomedin 4	OLFM4	150.67	3542.76
7058	Thrombospondin 2	THBS2	163.66	24.03
115908	Diffuse panbronchiolitis critical region 1	CTHRC1	174.61	1204.56
4680	Collagen triple helix repeat containing 1	CEACAM6	203.78	2542.02
3624	Inhibin beta A subunit	INHBA	219.12	368.69
1290	Collagen type V alpha 2 chain	COL5A2	230.72	1064.68
54829	Asporin	ASPN	255.15	237.05
1366	Claudin 7	CLDN7	288.09	356.71
