# Supplementary material for: Gastric Cancer Associated Genes Identified by an Integrative Analysis of Gene Expression Data
Source: Biomed Res Int. 2017 Jan 23;2017:7259097. doi: 10.1155/2017/7259097 (PMC5292384; doi:10.1155/2017/7259097)
Supplement: Supplementary file 1 — Table S1: Information for differentially expressed genes. [file 7259097.f1.pdf]

Table S1:Information for differentially expressed genes

EntrezID	Name	CombinedRP	AveLogFC	Pval
5225	PGC	22.37618865	14454.4758	0
57016	AKR1B10	22.86226955	6705.557765	0
9992	KCNE2	36.08428177	4314.327905	0
284340	CXCL17	49.18093279	3880.567262	0
135656	DPCR1	55.94229823	1892.035059	0
51208	CLDN18	57.3490307	3435.259165	0
1293	COL6A3	59.02243551	-3600.958417	0
1278	COL1A2	62.06096885	-3576.205929	0
3512	IGJ	62.80941701	25978.0491	0
1510	CTSE	64.51216896	4631.280569	0
340547	VSIG1	69.66145697	1752.542251	0
4499	MT1M	100.5789863	6787.140047	0
6947	TCN1	102.9607134	3564.358317	0
768239	PSAPL1	119.7813568	716.9719191	0
148808	MFSD4	143.5568227	1677.560594	0
10562	OLFM4	150.666316	-3542.760536	0
7058	THBS2	163.6563202	-24.03016606	0
4496	MT1H	167.5049606	7238.419671	0
115908	CTHRC1	174.6147569	-1204.55627	0
218	ALDH3A1	182.2048868	686.0077727	0
3485	IGFBP2	182.8744446	13742.24761	0
3490	IGFBP7	186.014491	34075.26854	0
51316	PLAC8	188.2023566	2091.586406	0
6750	SST	191.8200648	924.4507777	0
563	AZGP1	200.2215467	2030.55507	0
4680	CEACAM6	203.7822599	-2542.020962	0
1E+08	LOC100128893	205.7947701	721.3237822	0
361	AQP4	213.4236361	381.7968368	0
4477	MSMB	215.5581823	368.5352109	0
3624	INHBA	219.1191635	-368.6945035	0
115019	SLC26A9	221.4676235	507.0154925	0
1290	COL5A2	230.7153285	-1064.684233	0
4118	MAL	235.2413774	563.5230939	0
871	SERPINH1	243.2433433	1303.849567	0
54829	ASPN	255.1487608	-237.0489505	0
94234	FOXQ1	258.025839	2037.743693	0
1300	COL10A1	259.1455696	142.5328954	0
388743	CAPN8	263.2566507	892.3456491	0
2191	FAP	263.5453973	325.3248559	0
83641	FAM107B	268.4392172	1693.368707	0
151556	GPR155	273.2188057	7056.514842	0

1051	CEBPB	274.7527146	1469.58243	0
51738	GHRL	278.1721779	711.0985409	0
1366	CLDN7	288.091328	-356.7123433	0
1160	CKMT2	288.4004573	480.0402009	0
3397	ID1	289.7714528	8818.451709	0
80117	ARL14	298.3392322	637.3716819	0
7060	THBS4	300.587765	-2159.605376	0
10398	MYL9	302.4357263	-1624.112929	0
1301	COL11A1	307.753111	-200.7780742	0
2752	GLUL	314.0056913	997.2484898	0
7178	TPT1	317.0341531	22378.46889	0
6038	RNASE4	323.022807	1165.43795	0
6277	S100A6	323.2144571	2925.824666	0
11012	KLK11	323.5516353	908.1171728	0
4256	MGP	324.839462	4868.187762	0
9314	KLF4	327.7241393	1037.077622	0
3043	HBB	328.1106829	3729.291692	0
6281	S100A10	331.4706182	2393.424753	0
633	BGN	334.7641809	-488.4944655	0
80157	CWH43	343.1186487	66.06613347	0
8789	FBP2	343.4595549	197.7399521	0
7045	TGFBI	346.9860556	707.3673564	0
1562	CYP2C18	347.8602526	599.9397693	0
5873	RAB27A	347.9424335	704.2401073	0
2638	GC	362.1821298	2830.681529	0
4316	MMP7	364.1095701	5255.562378	0
1289	COL5A1	365.7385612	-637.6419615	0
166824	RASSF6	366.6317873	517.5700126	0
5176	SERPINF1	370.0868974	194.7832118	0
7850	IL1R2	371.6686883	520.6878701	0
6696	SPP1	372.0992078	2411.297896	0
7009	TMBIM6	373.3980261	7400.765217	0
4246	SCGB2A1	380.8103872	182.6352179	0
145226	RDH12	387.9837516	237.8785805	0
27089	UQCRQ	389.5120332	2486.882075	0
5827	PXMP2	391.5122595	1008.958566	0
56937	PMEPA1	395.8150757	-62.22753302	0
51092	SIDT2	396.3767796	1047.291791	0
6135	RPL11	403.6727751	12273.77413	0
25924	MYRIP	407.0448727	692.0272796	0
11224	RPL35	413.1613432	7233.879526	0
2495	FTH1	414.2453924	13061.73601	0
3772	KCNJ15	416.7739259	501.9603381	0
1528	CYB5A	419.788193	4465.193627	0

3315	HSPB1	420.0690883	-285.1450513	0
1001	CDH3	421.8605855	-209.0090764	0
1E+08	CD24	424.6185776	12370.22622	0
3206	HOXA10	425.0689898	-136.9562623	0
3939	LDHA	432.4720559	-1785.338595	0
72	ACTG2	432.7795175	-73130.01133	0
608	TNFRSF17	438.9461199	595.6216124	0
3320	HSP90AA1	439.8045153	2049.914531	0
5874	RAB27B	443.4152464	396.8587261	0
1508	CTSB	444.222131	2833.606659	0
6170	RPL39	444.3414818	30555.13675	0
11261	CHP	445.1447612	3974.466463	0
567	B2M	446.3136934	14814.68772	0
10410	IFITM3	447.2490932	6355.584436	0
6414	SEPP1	452.5851874	5411.334359	0
23673	STX12	457.0556232	765.3947708	0
2266	FGG	467.2960973	5549.107508	0
6223	RPS19	472.1547307	13891.28448	0
8527	DGKD	474.9579215	665.7060417	0
9528	TMEM59	477.7260607	3931.566259	0
10417	SPON2	479.2822528	1182.569186	0
4190	MDH1	479.3161294	1078.788032	0
4837	NNMT	480.117735	2341.231258	0
6175	RPLP0	481.4288327	9694.367575	0
229	ALDOB	481.8952428	12321.35332	0
4637	MYL6	483.7620954	-5402.915863	0
1015	CDH17	484.5930035	-560.8043541	0
54843	SYTL2	489.6934599	674.1140158	0
347902	AMIGO2	495.7421904	-634.5196665	0
716	C1S	496.5487763	6679.410438	0
56605	ERO1LB	496.6806875	514.1477214	0
131	ADH7	497.3864425	51.34773705	0
2243	FGA	501.1460843	850.1670341	0
79589	RNF128	503.1161747	638.4356676	0
768211	RELL1	503.5513347	963.535226	0
55450	CAMK2N1	510.2878213	-54.43502851	0
121512	FGD4	511.8432571	472.2547446	0
4312	MMP1	512.0460389	-573.4642993	0
23588	KLHDC2	517.1190861	1203.782717	0
5068	REG3A	517.6448362	616.8680019	0
3576	IL8	520.5120194	-289.5853247	0
81610	FAM83D	523.622869	-3000.498013	0
4582	MUC1	530.9815269	659.149953	0
2697	GJA1	532.8309415	-1258.957948	0

6822	SULT2A1	533.7645559	484.6713724	0
114132	SIGLEC11	535.4573469	197.6279075	0
6136	RPL12	537.3100488	19988.43346	0
7314	UBB	537.6338135	7925.213429	0
1164	CKS2	543.6452358	-560.5486025	0
1295	COL8A1	544.6490004	-256.2226424	0
8801	SUCLG2	544.9432642	637.6902076	0
6147	RPL23A	544.9969496	14376.48677	0
6695	SPOCK1	547.6738891	-254.6791322	0
285016	FAM150B	549.2025319	1146.882966	0
9076	CLDN1	550.4484326	1082.605353	0
1292	COL6A2	554.5627944	-486.304655	0
9235	IL32	554.8033939	1554.998766	0
1915	EEF1A1	554.9880527	10627.67369	0
80310	PDGFD	560.4664486	847.3630009	0
2982	GUCY1A3	563.783979	-303.8166043	0
10549	PRDX4	567.330684	1999.029099	0
25824	PRDX5	569.0631721	1871.294541	0
3373	HYAL1	577.7981241	652.9896696	0
9061	PAPSS1	581.4469707	1041.807714	0
754	PTTG1IP	582.1555399	2016.36066	0
220	ALDH1A3	586.281563	-306.5986944	0
506	ATP5B	587.2689507	684.0164387	0
203068	TUBB	587.8101431	-67.733311	0
3067	HDC	591.653372	196.3660918	0
12	SERPINA3	594.0070341	489.5073689	0
6231	RPS26	596.7083291	-7970.234477	0
1306	COL15A1	602.6419242	-804.8826773	0
1303	COL12A1	603.7260847	-127.2149502	0
5901	RAN	606.5576628	-641.5266736	0
340542	BEX5	608.1913019	482.2477954	0
5268	SERPINB5	608.6081255	-461.5729922	0
5688	PSMA7	608.9073213	-605.6383492	0
7805	LAPTM5	609.105659	317.5209189	0
4105	MAGEA6	612.5515374	-372.0433189	0
5376	PMP22	615.6648362	-899.7382728	0
5217	PFN2	618.4966946	-655.341003	0
6282	S100A11	619.8729795	1214.818917	0
400961	PAIP2B	623.8571859	241.8152468	0
3032	HADHB	624.2611539	524.2481584	0
22841	RAB11FIP2	627.7307223	689.1976789	0
3769	KCNJ13	629.2357802	65.57358268	0
9052	GPRC5A	630.6377972	-586.3658741	0
7388	UQCRH	635.6053513	2329.100917	0

2876	GPX1	636.9383058	4220.881765	0
51330	TNFRSF12A	637.8529314	280.418898	0
27346	TMEM97	640.3026346	1107.968837	0
7164	TPD52L1	641.0563256	576.7148473	0
79602	ADIPOR2	641.7049233	923.44766	0
7494	XBP1	642.5404559	2930.416229	0
7381	UQCRB	643.6983284	-1508.321146	0
7504	XK	645.6849884	111.0893718	0
7348	UPK1B	651.9639687	121.2561484	0
3091	HIF1A	654.1493096	-537.1306774	0
645	BLVRB	654.442454	2536.083697	0
2331	FMOD	654.8919742	2952.822195	0
29785	CYP2S1	657.7272588	313.5897669	0
3399	ID3	659.0178667	1949.88697	0
3983	ABLIM1	663.1007491	958.7692619	0
800	CALD1	666.1359392	-14695.84752	0
342979	PALM3	668.5240999	468.9280921	0
51643	TMBIM4	669.9112816	1892.539595	0
710	SERPING1	670.014325	-198.3287254	0
3815	KIT	670.9898022	244.3712323	0
9445	ITM2B	671.9030053	7564.105893	0
9370	ADIPOQ	672.2337814	36.72015699	0
4128	MAOA	672.4373825	-422.468635	0
1525	CXADR	674.7717385	473.2061117	0
549	AUH	675.7516807	826.7202928	0
3217	HOXB7	676.9595264	-203.1237458	0
4638	MYLK	680.7558556	-15760.70455	0
5919	RARRES2	682.9104216	324.0049998	0
4653	MYOC	684.6205536	-749.9960257	0
5757	PTMA	685.3384064	4579.435252	0
3418	IDH2	685.6594214	598.4709836	0
3303	HSPA1A	690.4867029	36637.79629	0
9636	ISG15	690.8301244	-373.3227409	0
1066	CES1	698.5376331	1975.526842	0
5034	P4HB	700.0102251	1922.430324	0
80781	COL18A1	705.0355501	5393.004338	0
1832	DSP	708.0471351	2208.919361	0
6156	RPL30	708.110651	20599.84919	0
579	NKX3-2	708.2081549	-48.73020456	0
9317	PTER	708.5374568	299.1283413	0
270	AMPD1	710.9246953	179.3567458	0
4697	NDUFA4	715.221186	-6682.527866	0
183	AGT	715.6079904	11571.93035	0
79888	LPCAT1	716.2354338	-383.7722813	0

5420	PODXL	718.6965932	1122.882571	0
27230	SERP1	718.7045299	893.7424997	0
60	ACTB	718.9840087	8383.282722	0
5396	PRRX1	721.5056773	-80.5966093	0
196	AHR	722.1541094	-391.876504	0
7033	TFF3	723.1583837	-61.7182915	0
10954	PDIA5	723.3078308	760.9666799	0
2981	GUCA2B	725.0823281	100.3322312	0
113146	AHNAK2	725.090497	-285.3496309	0
51660	BRP44L	731.4400681	1682.382204	0
151887	CCDC80	734.48086	-365.2355183	0
28958	CCDC56	735.9302726	831.8097221	0
84417	C2orf40	739.2546522	-16230.90205	0
10140	TOB1	745.3733888	726.2039227	0
57533	TBC1D14	746.1295362	843.5378404	0
80335	WDR82	747.62766	-397.2463258	0
7123	CLEC3B	749.1277736	1432.46678	0
306	ANXA3	749.5681369	875.9972885	0
7385	UQCRC2	753.5001627	64.30724317	0
3912	LAMB1	753.8856115	2639.143845	0
50	ACO2	754.7110616	263.2972731	0
1831	TSC22D3	756.5697257	3674.012712	0
10103	TSPAN1	760.0683748	289.6830453	0
55353	LAPTM4B	764.1323996	-1883.534523	0
10598	AHSA1	764.9579485	-456.4691103	0
54959	ODAM	765.3011315	266.8480643	0
60559	SPCS3	765.8499723	717.4793684	0
8673	VAMP8	771.0238651	3247.96076	0
2512	FTL	771.6559754	5767.883677	0
471	ATIC	782.6552144	-346.0874615	0
2950	GSTP1	784.0696936	2859.571765	0
2181	ACSL3	784.4897593	524.085617	0
8483	CILP	785.4091152	-667.3496366	0
522	ATP5J	786.1984783	-807.8196593	0
8991	SELENBP1	786.3676675	-3178.41571	0
1846	DUSP4	786.8086817	514.6000544	0
5160	PDHA1	787.2730037	485.8972437	0
57181	SLC39A10	789.0220875	-193.9717559	0
388677	NOTCH2NL	791.6780793	296.0874966	0
7153	TOP2A	794.7885664	-215.8277749	0
51495	PTPLAD1	794.9491082	1198.281458	0
2821	GPI	796.995414	141.8361205	0
10962	MLLT11	797.3539708	-568.2628206	0
6194	RPS6	797.4240666	26457.36397	0

4717	NDUFC1	801.8536435	500.9654324	0
11335	CBX3	803.1130226	-84.22012761	0
3158	HMGCS2	804.9258631	73.22661622	0
9111	NMI	808.7980683	-154.5735232	0
1373	CPS1	809.5757293	1982.857231	0
139341	FUNDC1	809.6734824	423.6750965	0
54504	CPVL	810.2027476	-132.3276037	0
55256	ADI1	811.7199157	1152.177064	0
114926	C8orf40	812.7007285	841.5867479	0
89894	TMEM116	812.8832159	359.3547087	0
8667	EIF3H	813.8702159	4755.702335	0
6124	RPL4	814.5193017	5413.519596	0
51776	ZAK	814.9611668	-3558.477431	0
6203	RPS9	815.6730036	6700.861855	0
5908	RAP1B	815.9283776	929.0061118	0
5328	PLAU	816.8595205	-37.7756464	0
3326	HSP90AB1	819.3698179	72.35440659	0
23603	CORO1C	819.9751529	-185.8423494	0
6161	RPL32	822.7121922	27383.29953	0
153572	IRX2	823.856991	125.6200773	0
154	ADRB2	824.0368165	157.2646443	0
3553	IL1B	824.1573756	570.6283205	0
27250	PDCD4	824.710916	926.0180729	0
2706	GJB2	825.899742	-393.5240862	0
3339	HSPG2	826.6930691	138.9586681	0
54460	MRPS21	829.2118015	159.0814561	0
5067	CNTN3	831.0881495	108.2640618	0
5476	CTSA	831.2855531	152.6932146	0
2122	MECOM	831.6856489	249.4948779	0
8815	BANF1	831.8858757	-96.95174472	0
83879	CDCA7	833.5654777	-259.6740196	0
7169	TPM2	834.4946941	-46339.1223	0
415116	PIM3	835.005248	2399.100759	0
3241	HPCAL1	836.8035534	327.3920835	0
6208	RPS14	837.297351	6076.320968	0
2330	FMO5	838.4924659	1123.762648	0
56889	TM9SF3	839.4737934	709.6555716	0
2	A2M	839.9324724	5565.153775	0
2162	F13A1	840.8937736	589.8763352	0
6632	SNRPD1	841.3775379	-132.9100306	0
55273	TMEM100	845.0089554	60.29406142	0
643201	LOC643201	845.2087583	5.237510926	0
6204	RPS10	847.1434438	6596.371833	0
144501	KRT80	852.1574487	-12.14646808	0

7534	YWHAZ	852.3227622	416.6490041	0
9833	MELK	852.4714765	-182.6467002	0
5436	POLR2G	852.5115172	904.8064173	0
284996	RNF149	852.9155244	217.8622592	0
5997	RGS2	853.2761543	-15949.52541	0
54979	HRASLS2	856.9788941	168.722598	0
7941	PLA2G7	857.8722872	-1.606095229	0
643911	CRNDE	858.0622585	268.2811436	0
11337	GABARAP	858.3920331	3796.052461	0
6634	SNRPD3	858.7125514	-301.4739233	0
1179	CLCA1	860.2447011	-106.8198438	0
91689	C22orf32	860.9346186	405.1386011	0
1909	EDNRA	861.032325	-257.7409433	0
8407	TAGLN2	861.718201	671.692979	0
4314	MMP3	861.9369424	-207.0515961	0
10528	NOP56	862.0697805	-172.3406602	0
58515	SELK	862.2451915	1248.298037	0
9843	HEPH	863.9771606	-436.0263069	0
51635	DHRS7	865.5554398	525.2662175	0
2810	SFN	871.2091575	-1113.002914	0
10134	BCAP31	871.8231137	811.3779406	0
25937	WWTR1	871.973107	-241.7550731	0
4716	NDUFB10	872.677807	588.6293924	0
84513	PPAPDC1B	872.8993833	487.3477586	0
1307	COL16A1	873.1316656	-650.035432	0
10809	STARD10	874.5578522	477.1707912	0
50507	NOX4	875.2750956	-15.37278559	0
9550	ATP6V1G1	875.627398	1764.738152	0
1123	CHN1	876.2985419	-168.4148069	0
26064	RAI14	879.333047	-256.8382445	0
10951	CBX1	880.1091268	-331.0893295	0
222008	VSTM2A	880.1668249	30.56719728	0
26872	STEAP1	880.4258383	-227.3604291	0
6144	RPL21	883.7331069	7502.27137	0.000265957
4282	MIF	884.9973705	4378.764144	0
5349	FXYD3	886.5810634	248.7334646	0.00026455
2203	FBP1	887.8162324	2313.807941	0.000263158
91137	SLC25A46	888.4087543	315.1263276	0.00026178
4601	MXI1	888.5167678	347.2551588	0.000260417
9358	ITGBL1	890.368068	190.1185921	0
9509	ADAMTS2	892.6588843	-127.5708017	0
405753	DUOXA2	892.9358794	170.9664183	0.000259067
4318	MMP9	897.9781189	-24.12037964	0
1.01E+08	OCLN	899.0743322	278.6140737	0.000257732

4677	NARS	899.889028	689.5699	0.00025641
153769	SH3RF2	901.7847416	126.1230912	0.000255102
25984	KRT23	902.6659214	-3.135260703	0
8972	MGAM	902.7992381	60.95854744	0.000253807
200879	LIPH	903.8091023	127.5206791	0.000252525
5159	PDGFRB	904.37333	2873.077485	0
999	CDH1	904.8581995	2251.270191	0.000251256
8507	ENC1	911.1255529	-253.3300948	0
493869	GPX8	911.4523038	-118.5822343	0
51280	GOLM1	913.3065122	381.6932698	0.00025
27087	B3GAT1	913.5172378	182.5054232	0.000248756
54205	CYCS	915.5728889	366.90232	0.000247525
80307	FER1L4	915.819573	390.3711965	0.000246305
55286	C4orf19	916.123124	333.9117224	0.000245098
285148	IAH1	918.0487519	-126.2676797	0
7852	CXCR4	925.3387147	15191.23122	0
26022	TMEM98	925.4839465	977.8828553	0.000243902
6158	RPL28	925.585267	5100.184905	0
79782	LRRC31	926.2572124	70.66341749	0.000242718
7168	TPM1	926.5686498	-6009.138998	0
90355	C5orf30	926.9254814	162.8585915	0.000241546
8904	CPNE1	927.3119535	-173.6671389	0
9245	GCNT3	928.0532346	-445.3906057	0
38	ACAT1	930.9652862	347.0240515	0.000240385
10057	ABCC5	931.4455648	260.7170003	0.000478469
5621	PRNP	933.4053663	-1534.942876	0
312	ANXA13	935.3099958	771.2474042	0
23231	SEL1L3	940.3690541	2059.060648	0.000473934
284578	LOC284578	943.7960446	29.29640411	0.000471698
2615	LRRC32	944.2210787	692.7678251	0
50808	AK3	944.4480208	575.9060694	0.000469484
2852	GPER	944.7226167	161.9801917	0.00046729
25994	HIGD1A	945.7524334	-26.01251971	0.000465116
10576	CCT2	950.5482786	310.6953024	0
3105	HLA-A	953.1340916	7240.670934	0.000462963
1434	CSE1L	954.3320553	-92.15507054	0
64094	SMOC2	956.3710599	-14711.66068	0
65987	KCTD14	958.1667511	184.7130058	0.000460829
23022	PALLD	958.611488	-3773.207259	0
5909	RAP1GAP	960.1073794	297.7139094	0.000458716
10808	HSPH1	960.1770537	4184.804002	0
51372	CCDC72	960.5473565	-541.0636843	0
6137	RPL13	961.2511661	18015.02133	0
5655	KLK10	962.3687349	-82.00602354	0

28996	HIPK2	962.8048368	247.6064331	0.000456621
85377	MICALL1	962.9657385	271.269677	0.000454545
84102	SLC41A2	963.1493263	202.2898995	0.000452489
253012	HEPACAM2	963.6517028	-120.7530148	0.00045045
144363	LYRM5	963.9373961	293.454979	0.00044843
4547	MTTP	964.2726388	261.8123234	0.000446429
112609	MRAP2	964.457493	144.0842377	0.000444444
1075	CTSC	964.9219665	-324.4352982	0
2983	GUCY1B3	965.33526	-147.8674376	0
51454	GULP1	969.8179251	188.3332707	0.000442478
25847	ANAPC13	971.5666202	466.9880453	0.000440529
4953	ODC1	972.8375701	-1123.872231	0
728342	LOC728342	974.2585146	14.06874566	0.000438596
3671	ISLR	974.8094188	2614.139522	0
1734	DIO2	975.2248757	-171.5539648	0
30008	EFEMP2	975.6725147	167.1925238	0
3627	CXCL10	977.2695949	-397.9592289	0
5111	PCNA	977.9500944	-189.8682633	0
8664	EIF3D	978.9345889	1517.938033	0.00026178
2877	GPX2	979.3165357	641.2589994	0.000436681
7851	MALL	980.5664757	-150.2165949	0.000260417
25874	BRP44	980.6851187	439.8174871	0.000434783
10040	TOM1L1	981.3819375	419.6587537	0.0004329
5478	PPIA	981.7759025	1256.138952	0.000515464
57758	SCUBE2	982.4601655	122.8993599	0.000431034
5734	PTGER4	982.8530591	640.6894934	0.000429185
7130	TNFAIP6	985.0179063	-74.77151022	0.000512821
5105	PCK1	985.5434454	786.6965648	0.00042735
2919	CXCL1	987.1922191	306.76434	0.000510204
55969	C20orf24	988.5004365	115.1593084	0.000507614
5646	PRSS3	989.1353262	-330.2191905	0.000505051
26509	MYOF	992.0621365	-281.2519316	0.000502513
11341	SCRG1	992.6565663	-1692.340658	5.00E-04
51146	A4GNT	995.8711692	157.2851394	0.000425532
4171	MCM2	996.3910352	-242.5953563	0.000497512
158038	LINGO2	997.0140525	72.77706115	0.000423729
891	CCNB1	1000.956784	-124.2327375	0.00049505
51186	WBP5	1000.976771	792.3185763	0.000421941
9349	RPL23	1001.260759	18783.28963	0.000492611
3312	HSPA8	1002.261288	-456.6499884	0.000735294
4946	OAZ1	1002.449097	10238.05352	0.000420168
5996	RGS1	1003.212966	2884.154403	0.000731707
7165	TPD52L2	1003.924736	-363.4174072	0.000728155
7378	UPP1	1005.522457	-145.7132854	0.000724638

9728	SECISBP2L	1005.533575	290.1091534	0.00041841
7529	YWHAB	1005.917024	1134.193136	0.000721154
705	BYSL	1010.188703	-61.24008931	0.000717703
5825	ABCD3	1010.332426	322.5596462	0.000416667
5660	PSAP	1011.156613	-168.2010152	0.000714286
10577	NPC2	1011.523916	4868.179611	0.0007109
8399	PLA2G10	1011.700186	80.53019186	0.000414938
7538	ZFP36	1011.730605	7614.625401	0.000413223
3799	KIF5B	1013.284414	-394.977785	0.000707547
4071	TM4SF1	1013.49218	3029.958356	0.000704225
822	CAPG	1013.675682	-145.3475641	0.000700935
10574	CCT7	1014.448697	262.7049484	0.000697674
128218	TMEM125	1015.049863	547.4891889	0.000411523
4969	OGN	1017.542112	-567.5185673	0.000694444
6635	SNRPE	1020.325233	49.67190182	0.000691244
84958	SYTL1	1022.097087	547.0077138	0.000409836
25837	RAB26	1022.416113	218.2396705	0.000408163
404037	HAPLN4	1022.815255	21.25177283	0.000406504
5627	PROS1	1023.045642	715.5034444	0.000688073
644150	WIPF3	1024.019754	128.0940348	0.000404858
55902	ACSS2	1025.018355	268.4573027	0.000403226
1975	EIF4B	1027.989015	1504.285637	0.000401606
55379	LRRC59	1029.448312	550.3659029	4.00E-04
53339	BTBD1	1029.454216	292.3815599	0.000684932
10979	FERMT2	1030.311256	-1338.210328	0.000681818
2110	ETFDH	1030.536101	78.28616465	0.000398406
3915	LAMC1	1031.801536	-1737.47252	0.000678733
727	C5	1031.942731	572.149948	0.000396825
3134	HLA-F	1036.304795	4752.576489	0.000675676
29108	PYCARD	1036.916476	999.4170375	0.000672646
5162	PDHB	1038.122472	442.3995619	0.000592885
55512	SMPD3	1040.988859	594.4679008	0.000590551
201895	C4orf34	1043.973685	390.029845	0.000588235
9837	GINS1	1045.897516	-170.9751652	0.000892857
23582	CCNDBP1	1047.108777	461.6081804	0.000585938
586	BCAT1	1047.431682	-37.23170683	0.000888889
114907	FBXO32	1048.625521	-2941.982521	0.000884956
103910	MYL12B	1050.056135	5974.211726	0.000881057
6168	RPL37A	1050.789853	12308.91144	0.000583658
29089	UBE2T	1053.830134	-121.5373246	0.001096491
7298	TYMS	1054.110369	-169.5191769	0.001091703
85477	SCIN	1054.96089	79.90404764	0.000775194
116535	MRGPRF	1056.545044	-331.0150961	0.001082251
6636	SNRPF	1058.73553	176.6446909	0.001508621

54812	AFTPH	1060.263595	594.6768345	0.000772201
255027	MPV17L	1060.42506	202.2886053	0.000769231
117247	SLC16A10	1064.441174	152.4887021	0.000766284
5446	PON3	1064.921305	3283.16556	0.000763359
291	SLC25A4	1065.070788	-100.0502341	0.000760456
2139	EYA2	1065.218551	72.50985612	0.000757576
2354	FOSB	1065.442169	1798.928564	0.000754717
8490	RGS5	1066.11922	-2833.52961	0.00075188
23601	CLEC5A	1066.280597	14.66028349	0.001495726
1350	COX7C	1066.647959	-2375.29597	0.000749064
6129	RPL7	1067.561319	2456.851065	0.000746269
10874	NMU	1067.691756	-30.72461391	0.001489362
5692	PSMB4	1068.593326	2348.265045	0.001483051
339	APOBEC1	1070.258638	60.04415783	0.000743494
2048	EPHB2	1070.67276	-44.26502344	0.001476793
57153	SLC44A2	1071.656977	342.9598054	0.000740741
1942	EFNA1	1073.829343	6.412630489	0.001680672
1033	CDKN3	1075.829288	-175.8654463	0.00167364
8864	PER2	1076.023608	140.3696184	0.000738007
8884	SLC5A6	1077.98522	-61.75535846	0.001666667
27098	CLUL1	1078.805896	31.60394122	0.000919118
56994	CHPT1	1078.982964	228.2550965	0.000915751
7832	BTG2	1080.75345	-17003.9507	0.000909091
29127	RACGAP1	1083.618968	-342.0929556	0.002074689
2207	FCER1G	1085.515619	1421.349621	0.002066116
6119	RPA3	1085.93198	-4.296955044	0.002057613
83661	MS4A8B	1086.942336	42.57332789	0.000905797
2171	FABP5	1087.400242	608.147539	0.000902527
140628	GATA5	1088.613363	149.6731233	0.000899281
27249	MMADHC	1088.773843	474.8676633	0.000896057
521	ATP5I	1089.144921	159.2231024	0.000892857
5408	PNLIPRP2	1089.428553	46.80250942	0.00088968
2517	FUCA1	1093.019198	544.5978832	0.000886525
23530	NNT	1096.087238	213.9246835	0.000883392
412	STS	1096.11167	78.48737482	0.000880282
7837	PXDN	1096.527357	773.1184661	0.002254098
6234	RPS28	1097.57893	43928.48752	0.000877193
51303	FKBP11	1097.598837	1280.345469	0.000874126
23327	NEDD4L	1098.520606	158.777102	0.00087108
81611	ANP32E	1099.529778	-125.3198927	0.002244898
27324	TOX3	1100.107686	75.74905065	0.000868056
2571	GAD1	1102.128773	-41.95338401	0.002235772
1936	EEF1D	1102.205101	2777.416664	0.002226721
124944	C17orf49	1103.154564	523.5658152	0.002217742

91894	C11orf52	1106.340421	276.7404569	0.000865052
81	ACTN4	1108.34013	-76.24617491	0.002208835
7037	TFRC	1108.840043	-440.833656	0.0022
1736	DKC1	1109.904883	3.027930985	0.002390438
8763	CD164	1111.949854	394.5301655	0.001030928
22908	SACM1L	1112.346142	379.1540424	0.001027397
11226	GALNT6	1112.581914	166.360383	0.001023891
4111	MAGEA12	1112.873885	-127.1481141	0.002579365
2617	GARS	1113.750961	-309.6694519	0.00256917
51501	C11orf73	1116.055913	-394.0860933	0.002755906
4982	TNFRSF11B	1116.43199	112.8048668	0.002745098
8671	SLC4A4	1117.356903	1566.75424	0.001190476
1557	CYP2C19	1117.519557	410.8039242	0.001186441
90865	IL33	1118.649428	839.2971794	0.001182432
10365	KLF2	1118.883537	5429.64639	0.001178451
151230	KLHL23	1119.605724	-218.433172	0.002734375
10972	TMED10	1120.383864	1545.44806	0.001174497
1829	DSG2	1120.42761	260.4947199	0.001170569
57509	MTUS1	1120.57365	192.5200876	0.001166667
1973	EIF4A1	1121.405935	1003.164889	0.002723735
1545	CYP1B1	1123.377734	-523.083731	0.002713178
397	ARHGDIB	1124.761244	7901.639046	0.002895753
1351	COX8A	1126.052644	1110.031618	0.001162791
2562	GABRB3	1126.294438	80.30049225	0.00115894
8284	KDM5D	1126.33885	237.4101796	0.001155116
23090	ZNF423	1126.403642	-99.78657788	0.002884615
6506	SLC1A2	1127.065317	207.4166454	0.001151316
729	C6	1128.075185	689.8210319	0.001147541
10399	GNB2L1	1128.410217	3237.825753	0.001143791
886	CCKAR	1128.667657	103.757072	0.001140065
24137	KIF4A	1128.957478	-108.8134825	0.002873563
57168	ASPHD2	1129.300925	104.141151	0.001136364
79717	PPCS	1130.788302	339.5031977	0.001132686
4711	NDUFB5	1131.255141	666.3265333	0.001129032
55829	SELS	1131.606753	313.7354821	0.001125402
1019	CDK4	1132.070147	651.4805289	0.002862595
79041	TMEM38A	1133.534454	73.82573898	0.001121795
3015	H2AFZ	1135.567831	-619.6357971	0.003422053
51596	CUTA	1135.907073	4460.230246	0.001118211
3728	JUP	1137.092013	624.900104	0.003598485
80139	ZNF703	1140.517924	-154.2978904	0.003584906
79949	C10orf81	1142.130042	-33.73720909	0.003759398
5805	PTS	1144.060762	483.106419	0.00111465
647979	LOC647979	1145.137636	-394.8841727	0.003745318

55165	CEP55	1145.187756	-127.1474852	0.003731343
55600	ITLN1	1146.294395	2.087328559	0.001111111
3400	ID4	1146.405779	1344.604311	0.001107595
28959	TMEM176B	1147.491246	430.8457269	0.003717472
10552	ARPC1A	1148.573201	478.52682	0.001104101
11153	FICD	1150.029087	167.1377852	0.001100629
10924	SMPDL3A	1150.15562	401.5605993	0.001097179
9891	NUAK1	1153.184069	153.774958	0.003888889
1E+08	HOTAIR	1154.976717	4.625621833	0.003874539
10904	BLCAP	1156.140191	-420.8488265	0.003860294
9104	RGN	1156.144283	490.9997823	0.00140625
2888	GRB14	1156.16173	1043.557275	0.003846154
2641	GCG	1156.619604	36.53766334	0.001401869
9258	MFHAS1	1156.834982	-147.0888708	0.003832117
27243	CHMP2A	1157.7072	793.5260746	0.001397516
64283	RGNEF	1157.786601	118.5948135	0.001393189
10615	SPAG5	1158.597695	-74.38293149	0.003818182
84674	CARD6	1159.48963	557.6391357	0.00154321
5125	PCSK5	1163.391055	0.235331217	0.003985507
847	CAT	1163.952282	680.8778416	0.001846154
3281	HSBP1	1164.867183	-246.2314244	0.004151625
51704	GPRC5B	1165.973199	1016.109083	0.001840491
9451	EIF2AK3	1166.877858	274.3418181	0.001834862
23531	MMD	1167.20732	99.55929854	0.004136691
3611	ILK	1167.472625	-207.2844141	0.004121864
7644	ZNF91	1167.893748	252.3547443	0.001829268
8836	GGH	1169.159571	257.8966424	0.004464286
6920	TCEA3	1171.2061	301.1821235	0.001823708
857	CAV1	1171.454259	-2655.891264	0.004626335
4714	NDUFB8	1172.059662	-505.8454344	0.001818182
9791	PTDSS1	1172.915401	322.1217333	0.004609929
26278	SACS	1173.931501	-520.5348132	0.004770318
124	ADH1A	1175.224488	1312.042962	0.001963746
10590	SCGN	1175.700721	777.1684986	0.001957831
57326	PBXIP1	1176.78323	297.4460148	0.001951952
26528	DAZAP1	1176.839039	-92.06641082	0.004753521
56898	BDH2	1177.686175	178.9746493	0.001946108
5157	PDGFRL	1178.601353	-81.67025912	0.004736842
54981	C9orf95	1178.912119	558.888308	0.002089552
5267	SERPINA4	1178.958978	442.0353008	0.002083333
84191	FAM96A	1180.625645	774.7727374	0.002077151
10476	ATP5H	1181.83147	-160.6098332	0.002071006
157869	C8orf84	1182.225552	-1447.594945	0.002064897
1611	DAP	1183.090029	442.2071512	0.002205882

7078	TIMP3	1183.161366	4501.018769	0.004895105
145788	FLJ27352	1184.258754	175.9141536	0.002199413
5708	PSMD2	1185.140573	48.58203681	0.004878049
8364	HIST1H4C	1185.348433	3312.95395	0.005034722
1657	DMXL1	1186.25969	293.5685858	0.002192982
1503	CTPS	1186.324974	-140.3762627	0.005190311
2192	FBLN1	1186.721351	-827.1262582	0.005344828
260425	MAGI3	1186.855023	157.37178	0.002186589
3486	IGFBP3	1187.709891	3112.287236	0.00532646
23366	KIAA0895	1188.022288	151.1814725	0.002325581
27295	PDLIM3	1189.926849	-9047.307133	0.005479452
3460	IFNGR2	1190.286716	49.63286472	0.005460751
9211	LGI1	1190.608018	0.703097709	0.002463768
30011	SH3KBP1	1191.356999	-287.5175679	0.005442177
1191	CLU	1191.797602	1476.252636	0.002456647
399665	FAM102A	1193.612284	1581.545518	0.00259366
3920	LAMP2	1194.870485	904.3085914	0.002729885
3171	FOXA3	1196.573526	166.9914544	0.002722063
1959	EGR2	1197.379811	1291.262383	0.00559322
6347	CCL2	1198.330882	3984.690726	0.005574324
10473	HMGN4	1198.782068	-108.9112557	0.005555556
3301	DNAJA1	1199.524791	191.7957722	0.005536913
48	ACO1	1200.56347	38.12411146	0.002714286
10921	RNPS1	1201.551795	111.785189	0.005685619
81606	LBH	1203.111144	476.8202176	0.005833333
788	SLC25A20	1203.310941	215.220865	0.002706553
55872	PBK	1204.685942	-178.5003756	0.005980066
55789	DEPDC1B	1204.830876	-32.3844567	0.005960265
9890	LPPR4	1205.65807	-38.1396238	0.006105611
22919	MAPRE1	1208.026219	-794.4576349	0.006085526
4594	MUT	1208.208131	456.8167152	0.002982955
55500	ETNK1	1208.369436	252.9062279	0.002974504
4726	NDUFS6	1208.858947	-342.7793958	0.006229508
55651	NHP2	1209.60625	878.5210964	0.00620915
6209	RPS15	1210.12778	20289.377	0.002966102
1043	CD52	1210.64362	1865.24649	0.006188925
284119	PTRF	1210.698636	15548.5606	0.006168831
84894	LINGO1	1211.778805	-6.600672874	0.006148867
133121	ENPP6	1214.499143	28.33609405	0.003239437
63924	CIDEC	1214.69867	59.19582448	0.003230337
55365	TMEM176A	1215.939389	2340.061146	0.006129032
3021	H3F3B	1216.38368	912.2128678	0.006109325
388610	TRNP1	1218.440203	284.8085927	0.003361345
10158	PDZK1IP1	1218.524032	370.2625124	0.00349162

5264	PHYH	1218.685605	221.8440065	0.003481894
57801	HES4	1218.99723	1852.020753	0.006089744
64284	RAB17	1219.346392	695.3439619	0.003472222
55076	TMEM45A	1219.995271	169.0034439	0.006230032
7095	SEC62	1220.838104	294.1468281	0.003601108
55351	STK32B	1221.636739	55.067135	0.00359116
8835	SOCS2	1221.726157	-229.5373079	0.003581267
514	ATP5E	1222.643654	1203.915778	0.006528662
254428	SLC41A1	1223.88458	276.3461131	0.003846154
6354	CCL7	1224.791857	-11.87354708	0.006825397
5054	SERPINE1	1224.81769	-48.22737759	0.006803797
5872	RAB13	1227.79826	-313.0255865	0.006782334
7035	TFPI	1228.626276	509.3993532	0.006761006
10226	PLIN3	1231.365336	-140.9892003	0.006896552
64288	ZNF323	1234.389455	117.274995	0.004109589
6418	SET	1235.351887	404.5286402	0.00703125
10351	ABCA8	1235.379738	132.1175786	0.004098361
10673	TNFSF13B	1236.872625	-112.1520195	0.007165109
7171	TPM4	1238.256293	-342.393885	0.007142857
202181	LOC202181	1238.39278	100.8285026	0.004211957
219931	TPCN2	1239.038928	175.3729248	0.004200542
293	SLC25A6	1239.65258	3677.013694	0.004189189
9926	LPGAT1	1239.720531	-70.79404278	0.007120743
11040	PIM2	1240.098776	257.148786	0.004177898
4286	MITF	1242.202153	279.3188086	0.004435484
4217	MAP3K5	1242.357682	427.9184401	0.004423592
10493	VAT1	1244.014258	625.8081819	0.007253086
23258	DENND5A	1245.040232	45.30130393	0.007230769
951	CD37	1245.55103	136.5571649	0.007208589
9805	SCRN1	1246.579741	-565.6517361	0.007186544
23452	ANGPTL2	1246.659375	218.8572267	0.007164634
7057	THBS1	1246.750387	141.3480845	0.007142857
1044	CDX1	1248.66313	-93.46868526	0.007575758
5052	PRDX1	1252.928654	692.2361969	0.00755287
307	ANXA4	1253.103157	15899.46697	0.00753012
58191	CXCL16	1255.316994	-31.40420237	0.007507508
65108	MARCKSL1	1255.467192	367.9052989	0.00748503
51079	NDUFA13	1255.91418	253.5151959	0.007462687
158158	RASEF	1256.200787	230.0416938	0.004545455
94274	PPP1R14A	1256.256902	-780.9059101	0.007440476
1848	DUSP6	1258.067157	1037.274459	0.007418398
55612	FERMT1	1258.885829	-99.13414886	0.00739645
9790	BMS1	1259.954702	-128.0773106	0.007374631
643155	C5orf43	1262.304185	337.9064859	0.004920213

8710	SERPINB7	1262.506356	21.2992114	0.004907162
10487	CAP1	1262.531077	-284.1049108	0.0075
6134	RPL10	1262.978777	18268.88944	0.00489418
939	CD27	1263.527557	532.877645	0.005013193
430	ASCL2	1265.751865	28.98724343	0.007624633
55454	CSGALNACT2	1265.881522	-86.81650292	0.007602339
5191	PEX7	1266.654237	211.0411434	0.005263158
51015	ISOC1	1267.161585	-104.3033974	0.005380577
440	ASNS	1267.258519	258.1895002	0.005366492
55355	HJURP	1268.186861	-110.1789425	0.007580175
6197	RPS6KA3	1268.369955	291.1808229	0.00755814
150696	PROM2	1268.485094	75.15420876	0.00535248
6218	RPS17	1269.214659	21712.03512	0.005338542
2633	GBP1	1269.441507	-388.0603175	0.007803468
2646	GCKR	1269.692382	99.87622122	0.005324675
54869	EPS8L1	1270.449925	498.2271301	0.005310881
5694	PSMB6	1271.641874	24.86386162	0.008213256
158	ADSL	1271.653472	-231.2738718	0.008189655
129607	CMPK2	1271.926075	237.9144295	0.005555556
134147	CMBL	1273.813804	194.7641536	0.005541237
5357	PLS1	1273.997174	407.0819583	0.005526992
2992	GYG1	1274.941663	-770.8481681	0.008309456
26	ABP1	1275.997707	-203.9728801	0.008285714
9928	KIF14	1276.491758	-67.3147582	0.008262108
9569	GTF2IRD1	1277.312489	-378.4937637	0.008238636
7368	UGT8	1277.883341	145.9245961	0.005769231
10242	KCNMB2	1279.871317	38.35317201	0.00601023
23321	TRIM2	1282.416828	245.9076204	0.005994898
2524	FUT2	1282.785222	157.9500327	0.005979644
57688	ZSWIM6	1284.002953	107.238612	0.005964467
11014	KDELR2	1284.473311	961.3202752	0.005949367
5652	PRSS8	1285.308407	1873.29144	0.005934343
81037	CLPTM1L	1285.359524	397.8606672	0.005919395
3098	HK1	1285.515263	176.5762162	0.005904523
2180	ACSL1	1285.622241	355.1618338	0.005889724
5702	PSMC3	1286.047636	-166.5817242	0.008923513
1114	CHGB	1287.191532	85.89430661	0.005875
83468	GLT8D2	1290.091213	-23.05953388	0.009322034
148418	SAMD13	1290.891755	133.9278649	0.005985037
84886	C1orf198	1290.956698	1206.047856	0.009295775
22948	CCT5	1291.155298	-223.7099624	0.009269663
3620	IDO1	1291.368375	-173.7573412	0.009383754
129293	C2orf89	1293.601882	71.38938914	0.009497207
4241	MFI2	1294.597647	-41.81114697	0.009610028

4627	MYH9	1294.81731	3749.185255	0.009583333
9551	ATP5J2	1294.925367	689.7110849	0.006218905
3921	RPSA	1296.174603	4654.028035	0.006203474
401081	FLJ22763	1297.961309	35.30834399	0.006435644
8174	MADCAM1	1298.432972	133.2953188	0.006419753
51144	HSD17B12	1299.197783	496.9597506	0.006527094
23462	HEY1	1299.802894	-0.024127312	0.009972299
133	ADM	1299.994198	367.7186661	0.006633907
23303	KIF13B	1300.072537	763.7421198	0.006617647
1072	CFL1	1300.907557	-11.31985212	0.009944751
3108	HLA-DMA	1301.024399	1726.474223	0.006723716
375484	C5orf25	1301.454613	110.0502037	0.006707317
10159	ATP6AP2	1301.895315	-125.8277676	0.010055096
1629	DBT	1304.317513	230.547181	0.006934307
11332	ACOT7	1304.949539	-88.29722017	0.010714286
80004	ESRP2	1305.603123	296.4279986	0.006917476
10082	GPC6	1305.802499	-4.393889251	0.010684932
5352	PLOD2	1306.533385	-211.4205508	0.010928962
6993	DYNLT1	1307.875873	810.0091784	0.011035422
687	KLF9	1307.931401	599.4062015	0.006900726
23336	SYNM	1308.200312	-11782.97036	0.011005435
2921	CXCL3	1308.205009	201.9012954	0.006884058
284244	LOC284244	1310.477241	51.88817753	0.007349398
6210	RPS15A	1311.006183	15346.81156	0.007331731
18	ABAT	1314.169084	659.8320297	0.007553957
26001	RNF167	1314.554416	223.3657301	0.007535885
10153	CEBPZ	1314.910654	-52.40708212	0.011924119
1499	CTNNB1	1314.942111	-47.26593373	0.011891892
87178	PNPT1	1318.365214	-161.1411175	0.011994609
70	ACTC1	1319.682708	-191.3916291	0.011962366
83416	FCRL5	1320.935623	119.5706852	0.007637232
23270	TSPYL4	1321.170119	457.9605532	0.007619048
151246	SGOL2	1323.02346	-96.70109246	0.012198391
54101	RIPK4	1323.581227	549.6722968	0.007719715
171024	SYNPO2	1323.792848	-4143.593405	0.012299465
58527	C6orf115	1324.168189	740.2280072	0.012266667
253827	MSRB3	1324.217619	-5086.856604	0.012234043
403313	PPAPDC2	1325.940614	241.0708466	0.007819905
1786	DNMT1	1327.791734	108.3076196	0.012334218
3169	FOXA1	1328.944931	328.3602658	0.008274232
29901	SAC3D1	1329.439927	-12.50479954	0.012301587
7639	ZNF85	1330.497249	209.5928693	0.008372642
1509	CTSD	1332.956939	241.104488	0.012401055
10265	IRX5	1333.353684	49.43479956	0.008920188

79902	NUP85	1334.570324	109.9283397	0.012631579
1603	DAD1	1335.722712	1580.6755	0.009250585
339168	TMEM95	1336.093234	7.699744763	0.009228972
6525	SMTN	1337.480761	-3417.923519	0.013254593
9260	PDLIM7	1337.896855	-205.7051095	0.013350785
8935	SKAP2	1339.481462	-339.357928	0.013315927
7247	TSN	1340.438861	17.78249706	0.013541667
90488	C12orf23	1340.729754	313.5579109	0.009207459
125704	FAM69C	1340.974914	25.8531383	0.009186047
6950	TCP1	1341.60603	-126.9217735	0.013636364
254295	PHYHD1	1343.602944	132.798941	0.00974478
51527	C14orf129	1343.955544	223.7307558	0.009722222
153562	MARVELD2	1344.380855	435.9192677	0.009815242
83449	PMFBP1	1345.770184	-0.866903077	0.013953488
8416	ANXA9	1346.220649	284.1923156	0.013917526
3479	IGF1	1346.710948	-152.6744248	0.014010283
11004	KIF2C	1347.178168	-84.38371061	0.013974359
79709	GLT25D1	1347.828386	-61.84324852	0.013938619
84316	LSMD1	1350.115825	190.2618803	0.014030612
84870	RSPO3	1351.35275	-898.0458677	0.013994911
9218	VAPA	1352.04952	287.3726775	0.010023041
51061	TXNDC11	1352.247263	461.2890538	0.01
10484	SEC23A	1352.382432	-888.0162961	0.014086294
22875	ENPP4	1353.215947	54.64370575	0.009977064
6935	ZEB1	1353.36817	-1286.057867	0.014177215
79070	KDELC1	1353.914761	-25.06355854	0.014141414
4605	MYBL2	1354.303824	-72.82265683	0.014357683
56954	NIT2	1354.668398	267.2097822	0.014447236
1486	CTBS	1356.663941	280.8397092	0.010297483
116372	LYPD1	1357.033225	18.10171752	0.014786967
2690	GHR	1358.030119	57.6400641	0.010273973
10650	SLMO1	1358.45228	-7.552011113	0.014875
259266	ASPM	1359.336436	-48.12637847	0.015087282
91624	NEXN	1360.208025	-328.5681996	0.015049751
10112	KIF20A	1360.874011	-86.4717844	0.015260546
57189	KIAA1147	1361.097531	384.6678897	0.010364465
414	ARSD	1361.123968	300.6681956	0.010340909
56670	SUCNR1	1361.36236	-26.35441113	0.015346535
57476	GRAMD1B	1361.971768	167.5405703	0.010430839
597	BCL2A1	1362.135913	114.6364564	0.015308642
9976	CLEC2B	1362.467898	428.0606231	0.015270936
1153	CIRBP	1363.237207	1035.953606	0.010746606
4782	NFIC	1366.479546	188.3125072	0.011060948
63976	PRDM16	1367.316566	139.1553526	0.011036036

64951	MRPS24	1367.429159	1320.70523	0.011011236
79153	GDPD3	1368.163764	144.7937977	0.010986547
10213	PSMD14	1369.67525	-185.3454618	0.016339066
6139	RPL17	1369.800042	11149.40521	0.011185682
118429	ANTXR2	1370.013218	-1083.665534	0.01629902
64859	OBFC2A	1370.976165	90.58729895	0.016259169
55048	VPS37C	1371.312438	332.2106553	0.011272321
6631	SNRPC	1372.524629	280.5899226	0.016463415
6464	SHC1	1373.023215	194.1220892	0.016423358
81669	CCNL2	1373.035568	-0.101389057	0.016383495
6159	RPL29	1373.053723	6820.052049	0.016464891
10969	EBNA1BP2	1375.001025	681.9380959	0.016545894
57124	CD248	1376.583236	-73.65949564	0.016987952
10105	PPIF	1376.67775	363.387885	0.011804009
10643	IGF2BP3	1376.935545	-44.77385094	0.016947115
9768	KIAA0101	1377.29768	-170.1677774	0.017026379
30061	SLC40A1	1378.770924	1029.855028	0.017105263
80114	BICC1	1379.640548	-87.52790192	0.017064439
23169	SLC35D1	1379.720084	118.1404461	0.012222222
127707	KLHDC7A	1380.145342	40.93983741	0.012305987
3727	JUND	1380.713615	246.5726645	0.012389381
9784	SNX17	1380.844339	287.8953421	0.017142857
4332	MNDA	1381.915247	156.5895518	0.017220903
25831	HECTD1	1383.418831	674.4065079	0.012472406
196463	PLBD2	1383.958953	23.41191325	0.017654028
930	CD19	1385.205347	519.7086932	0.012665198
51386	EIF3L	1385.291662	475.4188868	0.012637363
5327	PLAT	1386.395421	1898.451065	0.012609649
683	BST1	1386.424809	20.71126779	0.017730496
10279	PRSS16	1387.030739	95.36942914	0.012800875
6772	STAT1	1387.072345	178.7622312	0.017924528
7737	RNF113A	1387.178401	1447.071749	0.012772926
3738	KCNA3	1388.046492	196.1832076	0.012745098
9510	ADAMTS1	1389.802544	498.7933972	0.012717391
8842	PROM1	1390.058901	-37.67043564	0.018470588
6176	RPLP1	1391.175699	27249.96476	0.018661972
2151	F2RL2	1392.285584	-97.60865066	0.018852459
533	ATP6V0B	1393.278688	755.6149384	0.013232104
23158	TBC1D9	1394.052675	361.6497761	0.013203463
858	CAV2	1394.836153	-819.8741091	0.018808411
83939	EIF2A	1396.027194	298.9413578	0.013822894
4929	NR4A2	1396.584015	237.1778523	0.013793103
2149	F2R	1397.006134	-70.72072088	0.019114219
6146	RPL22	1398.20241	538.3426342	0.019186047

332	BIRC5	1398.448369	-64.9036726	0.019257541
11260	XPOT	1399.007968	-137.5898691	0.019212963
7431	VIM	1399.354861	31089.25053	0.019284065
58189	WFDC1	1400.758983	-437.1081046	0.014516129
4700	NDUFA6	1400.943095	-231.2180141	0.014484979
253558	LCLAT1	1401.523563	-73.26521971	0.019700461
7705	ZNF146	1402.50862	-187.6748066	0.019885057
2778	GNAS	1404.307628	4756.207833	0.014561028
1012	CDH13	1404.769957	209.9918586	0.020183486
64710	NUCKS1	1405.234901	-132.0928413	0.020251716
8470	SORBS2	1405.92393	228.3823567	0.014850427
27101	CACYBP	1406.303508	-59.14317772	0.020319635
256586	LYSMD2	1407.840962	276.8815038	0.02095672
1576	CYP3A4	1409.434148	2678.195309	0.015565032
113263	GLCCI1	1409.876592	469.8435539	0.015638298
1535	CYBA	1410.345545	64.53020765	0.021136364
221294	NT5DC1	1410.468463	217.2561679	0.015711253
54971	BANP	1411.158694	-92.81118303	0.021088435
50848	F11R	1411.885778	1402.211677	0.015783898
51514	DTL	1412.175692	-76.79372826	0.021266968
6167	RPL37	1412.607182	13469.12904	0.015856237
140609	NEK7	1413.969272	-82.90183805	0.021331828
55711	FAR2	1414.524929	-30.01057723	0.021283784
5707	PSMD1	1414.723743	-221.0823544	0.021235955
7277	TUBA4A	1416.276585	164.68756	0.021412556
9056	SLC7A7	1416.294816	303.9679353	0.021364653
2620	GAS2	1416.418752	191.6560013	0.016350211
2673	GFPT1	1420.124646	339.6972685	0.016947368
1178	CLC	1420.202447	72.39756072	0.016911765
1000	CDH2	1422.273667	1884.913766	0.017400419
64151	NCAPG	1422.879154	-58.85135756	0.022544643
145270	PRIMA1	1424.258279	-44.50751484	0.017364017
5496	PPM1G	1425.678576	-24.00925016	0.023385301
2195	FAT1	1426.161385	-50.17600789	0.023333333
25797	QPCT	1426.58057	47.38521341	0.023281596
10483	SEC23B	1427.58453	249.8604622	0.017954071
79101	TAF1D	1430.138742	36.16069452	0.023451327
6400	SEL1L	1431.520501	196.4096572	0.0184375
26751	SH3YL1	1431.535014	1817.510637	0.018399168
81621	KAZALD1	1433.799333	445.6675353	0.018672199
4692	NDN	1434.335499	-180.4531353	0.02384106
56925	LXN	1434.693433	3459.48915	0.023898678
10637	LEFTY1	1435.899135	575.842607	0.023956044
1843	DUSP1	1436.520754	-7062.935529	0.019047619

4666	NACA	1437.341315	12782.48401	0.01911157
9167	COX7A2L	1439.299996	924.6272068	0.019381443
91663	MYADM	1439.524657	301.8095098	0.024890351
7342	UBP1	1440.306146	-28.48475856	0.024835886
7311	UBA52	1441.513458	8657.665903	0.025
139596	UPRT	1443.044739	160.4172041	0.020164609
9446	GSTO1	1444.687356	853.0002373	0.025272331
10797	MTHFD2	1444.978516	36.86951476	0.025217391
360	AQP3	1447.316259	153.1486198	0.021516393
50865	HEBP1	1447.979764	1235.358855	0.021472393
3575	IL7R	1448.820787	2148.061532	0.025813449
27430	MAT2B	1449.280352	386.7794069	0.021734694
9144	SYNGR2	1449.459038	2039.792586	0.025757576
7280	TUBB2A	1449.934092	-127.8063694	0.025809935
6863	TAC1	1450.615003	-1.104659486	0.02575431
58508	MLL3	1450.726651	409.1773512	0.021995927
1063	CENPF	1450.879586	-48.47163592	0.025698925
5121	PCP4	1450.958803	-2063.825963	0.025751073
203100	HTRA4	1451.477832	-40.54942988	0.025910064
5355	PLP2	1452.822302	268.7286787	0.026495726
948	CD36	1453.826952	118.2456719	0.022256098
9086	EIF1AY	1453.846502	30.07687361	0.022210953
59084	ENPP5	1454.772678	138.6452533	0.022267206
1558	CYP2C8	1454.968594	249.2654475	0.022323232
164832	LONRF2	1457.720641	-380.691781	0.022635815
22983	MAST1	1458.287649	66.31275081	0.022791165
5245	PHB	1458.353842	-88.02765836	0.02782516
23710	GABARAPL1	1458.417703	-97.92084707	0.022745491
25977	NECAP1	1458.746877	145.7666007	0.0227
148811	PM20D1	1459.134112	52.80390162	0.022754491
10635	RAD51AP1	1459.420318	-116.5192114	0.028085106
84662	GLIS2	1460.014419	269.9226813	0.028025478
7262	PHLDA2	1460.59665	-9.943863766	0.028072034
545	ATR	1461.128798	148.4077125	0.028012685
64764	CREB3L2	1461.538831	554.8623455	0.023007968
4254	KITLG	1462.07704	193.1858074	0.02306163
5468	PPARG	1462.520613	-15.23881153	0.023115079
58528	RRAGD	1463.224001	-60.27113475	0.028270042
4710	NDUFB4	1464.023689	-1541.437852	0.023267327
169841	ZNF169	1464.292324	151.3363359	0.023221344
3688	ITGB1	1464.331106	-270.8962352	0.028315789
64981	MRPL34	1465.180322	693.8784905	0.023274162
23212	RRS1	1465.827788	-13.08403807	0.028466387
197370	NSMCE1	1466.553511	-163.6109512	0.028406709

51602	NOP58	1467.280016	1777.891304	0.028661088
338	APOB	1467.660782	1469.436834	0.023720472
79094	CHAC1	1467.97297	102.8915223	0.023772102
57475	PLEKHH1	1468.266607	113.2186189	0.02372549
220963	SLC16A9	1468.672164	42.5591136	0.023776908
5036	PA2G4	1468.729053	598.8735981	0.029227557
64816	CYP3A43	1469.120768	698.0012258	0.023828125
976	CD97	1469.122793	1133.20122	0.029166667
11135	CDC42EP1	1470.079753	4491.831635	0.023879142
3065	HDAC1	1472.174804	875.6607445	0.02962578
3178	HNRNPA1	1472.615715	2438.048488	0.024027237
25875	LETMD1	1472.995776	642.5962773	0.023980583
64318	NOC3L	1474.668323	39.83770503	0.02966805
8905	AP1S2	1474.770062	-216.1202779	0.029606625
6274	S100A3	1475.879906	-16.15630983	0.02964876
9900	SV2A	1475.902393	-47.71569352	0.029587629
7803	PTP4A1	1476.345771	22.48205603	0.024612403
29997	GLTSCR2	1476.576741	6231.860183	0.024564797
2519	FUCA2	1477.495068	-4.348139367	0.029835391
2647	BLOC1S1	1477.635384	393.2354635	0.024517375
57717	PCDHB16	1477.707616	-14.25952329	0.029774127
4973	OLR1	1477.853945	-9.196704293	0.029713115
92840	REEP6	1477.938505	485.365884	0.029591837
23554	TSPAN12	1478.315979	267.0771702	0.024759152
587	BCAT2	1478.71805	500.4569313	0.024807692
27289	RND1	1480.211093	115.7548402	0.025047985
23658	LSM5	1480.366557	92.85607143	0.030040733
865	CBFB	1481.212678	328.6908504	0.030081301
4640	MYO1A	1483.03439	31.11370997	0.025383142
9802	DAZAP2	1483.037056	2480.535208	0.025334608
375295	LOC375295	1483.408788	8.372316254	0.030769231
57493	HEG1	1484.415181	384.5919289	0.031111111
199713	NLRP7	1484.796731	52.01940039	0.025763359
79853	TM4SF20	1485.274813	-22.48156445	0.03125
9284	NPIP	1485.467049	-88.52935867	0.031488934
54874	FNBP1L	1487.001952	401.6066178	0.026380952
6745	SSR1	1487.09173	850.3366992	0.026425856
1340	COX6B1	1487.257531	980.1602497	0.031863727
55061	SUSD4	1488.176224	-5.665999097	0.026565465
54842	MFSD6	1488.89521	-87.78793117	0.026515152
998	CDC42	1489.304053	212.7069962	0.0319
2621	GAS6	1489.465643	239.0549506	0.026559546
780	DDR1	1490.081814	155.4390655	0.026792453
2108	ETFA	1490.935483	-44.9282727	0.027024482

4645	MYO5B	1491.462866	144.5852145	0.027067669
51162	EGFL7	1491.899098	592.696682	0.032335329
7347	UCHL3	1491.904059	-154.7426466	0.032270916
27335	EIF3K	1492.417091	180.9985603	0.03250497
6352	CCL5	1493.498746	235.1653462	0.032738095
10096	ACTR3	1493.867486	-178.2837323	0.032871287
10542	HBXIP	1495.785572	479.2491349	0.028330206
54915	YTHDF1	1495.85619	65.47241672	0.033234714
85462	FHDC1	1496.442741	112.6998113	0.028651685
57530	CGN	1496.95335	1110.011971	0.028691589
23196	FAM120A	1497.926782	356.7735616	0.02891791
3398	ID2	1498.689879	1697.773332	0.033759843
8540	AGPS	1498.756971	4.431965789	0.033791749
23204	ARL6IP1	1499.101209	137.769704	0.033823529
3069	HDLBP	1499.662705	519.2604105	0.029608939
23560	GTPBP4	1499.771543	-51.81645372	0.033953033
1287	COL4A5	1500.020725	-5875.195028	0.029553903
489	ATP2A3	1501.05513	152.5525795	0.029777365
6039	RNASE6	1501.226703	27.74827637	0.034375
11221	DUSP10	1501.239173	-1.732212066	0.034307992
4188	MDFI	1501.249735	95.08465715	0.034241245
290	ANPEP	1501.455937	-221.8006734	0.034174757
50509	COL5A3	1501.551125	-32.14550741	0.034108527
80223	RAB11FIP1	1501.772952	228.7159582	0.029814815
4330	MN1	1502.029587	-96.77834092	0.034139265
114908	TMEM123	1502.101214	1078.723378	0.034073359
10234	LRRC17	1502.59955	146.9343073	0.029944547
84293	C10orf58	1502.932327	-93.61210943	0.034489403
259239	WFDC11	1504.574188	4.823547862	0.030073801
4170	MCL1	1505.457297	482.631815	0.035
5139	PDE3A	1505.598666	-36.77621259	0.035028791
222643	UNC5CL	1506.711812	152.4873553	0.030294659
113130	CDCA5	1507.01405	-78.48534149	0.035153257
223	ALDH9A1	1507.343638	235.2365713	0.030183486
57405	SPC25	1508.396153	-62.52065934	0.035564054
54210	TREM1	1508.654334	-32.82998848	0.035687023
24146	CLDN15	1508.945121	-84.35827842	0.035619048
594	BCKDHB	1509.224917	120.2193087	0.030494505
9902	MRC2	1509.548947	2371.017792	0.035646388
6629	SNRPB2	1509.787837	193.7671699	0.035673624
10135	NAMPT	1510.597155	415.8143476	0.030530165
84918	LRP11	1512.171185	-216.0993366	0.036079545
8655	DYNLL1	1513.254053	-736.7462456	0.03610586
84327	ZBED3	1514.326409	762.9393257	0.031386861

890	CCNA2	1515.069371	-76.77847978	0.036320755
9136	RRP9	1515.455968	110.5776085	0.036252354
84933	C8orf76	1518.219603	-24.55855754	0.036842105
1580	CYP4B1	1518.406891	112.8108581	0.032240437
3918	LAMC2	1518.887736	19.63655983	0.036866792
677	ZFP36L1	1518.918709	-382.6985292	0.036797753
1471	CST3	1519.509304	1620.89871	0.032454545
11067	C10orf10	1519.82649	3153.147547	0.037196262
9352	TXNL1	1519.976569	536.9767233	0.032667877
1811	SLC26A3	1521.822328	6.517207718	0.032820976
9045	RPL14	1521.856101	18734.32032	0.032761733
2893	GRIA4	1522.346282	38.3528458	0.032702703
158405	KIAA1958	1523.040136	83.83475409	0.032733813
9493	KIF23	1523.416329	-28.64144107	0.037686567
63928	CHP2	1525.038832	-8.756660178	0.033034111
10418	SPON1	1526.367172	-1763.127057	0.037988827
84823	LMNB2	1526.903375	-94.15134699	0.038011152
1062	CENPE	1527.556692	-55.31440277	0.03812616
771	CA12	1527.880186	46.53528277	0.033781362
5999	RGS4	1528.562179	13144.44605	0.038425926
11100	HNRNPUL1	1528.950115	304.3509748	0.03844732
55737	VPS35	1529.664592	1126.939059	0.034168157
9231	DLG5	1530.059402	-316.9106794	0.038560886
65985	AACS	1530.245698	141.8516849	0.034107143
5094	PCBP2	1530.479176	1502.603138	0.034046346
5885	RAD21	1530.586861	192.0307492	0.038674033
11057	ABHD2	1530.660267	127.0027295	0.033985765
84722	PSRC1	1531.27396	-128.2503527	0.038878676
51301	GCNT4	1531.963659	10.05841666	0.034042553
1593	CYP27A1	1532.101429	3171.311407	0.038990826
128866	CHMP4B	1532.806599	-175.712092	0.039010989
55859	BEX1	1533.466461	485.3254659	0.034424779
56992	KIF15	1533.53864	-47.91724702	0.039213894
5595	MAPK3	1534.19569	165.2141766	0.034452297
9122	SLC16A4	1534.489317	627.1370702	0.039507299
7052	TGM2	1534.846657	-113.5533367	0.039526412
1111	CHEK1	1535.014567	-54.05190158	0.039454545
56906	THAP10	1536.413197	179.7950951	0.035097002
3689	ITGB2	1537.794681	1357.274383	0.040199637
123920	CMTM3	1538.658791	1308.876567	0.040307971
128153	SPATA17	1538.912028	4.893041979	0.040235081
5184	PEPD	1539.015157	1112.394929	0.040162455
55695	NSUN5	1539.930964	145.0471061	0.04036036
3119	HLA-DQB1	1540.970082	1113.214225	0.040467626

85437	ZCRB1	1541.79155	28.49059519	0.036707746
4259	MGST3	1541.871011	-123.3806414	0.036643234
23167	EFR3A	1542.928463	-247.5403378	0.041113106
6698	SPRR1A	1543.416795	33.80615395	0.036754386
389792	IER5L	1543.704228	137.4668945	0.041308244
10273	STUB1	1544.336559	891.9677666	0.03704028
6370	CCL25	1544.368325	-13.42881891	0.036975524
80328	ULBP2	1544.469529	-37.59300153	0.041413238
55608	ANKRD10	1544.517388	203.3709129	0.041339286
23228	PLCL2	1545.463007	131.8020864	0.037172775
64123	ELTD1	1546.186058	149.2734795	0.041800357
9369	NRXN3	1546.605428	-84.30734053	0.041725979
341640	FREM2	1547.722859	103.307824	0.037979094
55240	STEAP3	1548.125399	210.1995198	0.038173913
4000	LMNA	1550.22246	1086.28976	0.042717584
8237	USP11	1550.384567	337.4030233	0.042819149
375056	MIA3	1550.471365	200.548761	0.038888889
4436	MSH2	1550.54794	110.2680646	0.042743363
65065	NBEAL1	1550.924077	87.43604054	0.038908146
2523	FUT1	1551.21314	214.3193803	0.03884083
11272	PRR4	1551.373947	-28.76127836	0.042844523
51192	CKLF	1551.724305	164.4768103	0.042857143
283208	P4HA3	1552.614496	-24.12361553	0.042957746
5230	PGK1	1555.345222	521.7768414	0.043585237
51020	HDDC2	1555.600529	224.9813641	0.039982729
2872	MKNK2	1556.367139	2134.611853	0.04
9951	HS3ST4	1558.611288	60.7582049	0.040619621
51009	DERL2	1560.597638	382.9015028	0.041408935
953	ENTPD1	1560.741756	-156.6829722	0.045526316
8611	PPAP2A	1561.547601	-546.3519324	0.045621716
51110	LACTB2	1562.050984	-9.990525025	0.045629371
57447	NDRG2	1562.62377	5493.210577	0.042367067
5782	PTPN12	1563.521727	1027.661975	0.046160558
284266	SIGLEC15	1563.783609	-33.09173484	0.04255137
83935	TMEM133	1565.132926	368.9283144	0.042735043
348262	FAM195B	1566.009101	813.6630788	0.046689895
27020	NPTN	1566.146031	-490.1487005	0.046695652
79981	FRMD1	1566.433627	84.50777032	0.04334471
84518	CNFN	1566.869456	111.1118443	0.046875
92922	CCDC102A	1567.194891	79.29342054	0.046793761
1644	DDC	1567.678893	2.152732467	0.046712803
63027	SLC22A23	1569.335785	101.4227853	0.044037479
285761	DCBLD1	1570.134623	-65.57365432	0.047495682
285203	C3orf64	1570.641148	-160.6891944	0.047758621

1466	CSRP2	1572.549973	-5391.494814	0.04827883
83481	EPPK1	1575.651759	-73.09046566	0.048797251
2146	EZH2	1576.168749	20.80496015	0.04897084
285095	LOC285095	1576.97985	4.146830575	0.046604414
9223	MAGI1	1577.825704	131.0926481	0.046694915
93974	ATPIF1	1578.765118	1824.908847	0.047123519
7184	HSP90B1	1580.572978	6419.145765	0.047639123
10575	CCT4	1580.653651	1768.460647	0.049828767
7105	TSPAN6	1581.011694	778.2177899	0.047727273
9499	MYOT	1581.092004	-9.877374421	0.047647059
84504	NKX6-2	1581.312914	6.817338782	0.047651007
2274	FHL2	1581.336863	-895.5021136	0.04982906
